# Evaluating the protective effects of the Toll-like receptor (TLR) 21 ligand, CpG ODN, against necrotic enteritis in broiler chickens

**DOI:** 10.1371/journal.pone.0319404

**Published:** 2025-03-13

**Authors:** Mohammadali Alizadeh, Samson Oladokun, Charlotte Fletcher, Nitish Boodhoo, Fatemeh Fazel, Bahram Shojadoost, Sugandha Raj, Jiayu Zheng, Khaled Abdelaziz, Shayan Sharif

**Affiliations:** 1 Department of Pathobiology, Ontario Veterinary College, University of Guelph, Guelph, Ontario, Canada; 2 Department of Biochemistry and Biomedical Sciences, McMaster University, Hamilton, Ontario, Canada; 3 Ceva Animal Health Inc., Guelph, Ontario, Canada; 4 National Centre for Foreign Animal Disease, Winnipeg, Manitoba, Canada; 5 Clemson University School of Health Research (CUSHR), Clemson, South Carolina, United States of America; 6 Department of Animal and Veterinary Science, Clemson University, Clemson, South Carolina, United States of America; Cornell University, UNITED STATES OF AMERICA

## Abstract

Necrotic enteritis (NE), caused by *Clostridium perfringens* (*C. perfringens*), presents a challenge to the global broiler industry. Evidence suggests that Toll-like receptor (TLR) ligands can enhance the immune responses in chickens and protect them against infectious diseases. This study investigated the protective effects of TLR21 ligand class B CpG oligonucleotides (ODN) against NE in broiler chickens. On day 21 of age, chickens were injected with 50 or 100 μg CpG intramuscularly, and one group was injected with 50 μg CpG followed by a booster dose on day 22. Subsequently, birds were orally challenged with *C. perfringens* twice daily for three days, starting on day 22. On day 22, intestinal samples were collected for gene expression analysis. On day 25, all birds were euthanized, intestinal lesions were scored, and tissue samples were collected from the intestine for gene expression analysis, lymphocyte subset determination, and histomorphological analysis. Cecal contents were also collected for microbiome analysis. The results demonstrated that CpG pre-treatment, either at a single dose of 100 μg or two doses of 50 μg per bird, reduced lesion scores compared to the positive control. *C. perfringens* infection increased crypt depth in both the jejunum and ileum in the positive control group compared to both the CpG-treated group. At 22 days of age, CpG administration at doses of 100 μg per bird enhanced expression of TLR21, interleukin (IL)-2, CXCL8, IL-10, and interferon (IFN)-γ mRNA transcripts in both the jejunum and ileum. Additionally, at 25 days of age, the group pretreated with two doses of 50 μg of CpG per bird showed increased expression of all cytokines in both the jejunum and ileum compared to the control groups. The percentage of intestinal lymphocytes was not affected by CpG pre-treatment. However, CpG pretreatment at doses of 100 μg resulted in a higher abundance of the members of families *Lactobacillaceae* and *Bacteroidaceae*, which are crucial for maintaining gut health. In conclusion, our findings suggest that pretreatment of chickens with intramuscular administration of CpG may be effective in maintaining gut health during *C. perfringens* infection.

## Introduction

Necrotic enteritis (NE) is a multifactorial infectious disease that causes substantial economic losses to the poultry industry [1]. NE is characterized by overgrowth of *Clostridium perfringens* (*C. perfringens*) types A and C in the small intestines of broiler chickens aged 2-6 weeks [2]. Predisposing factors such as subclinical coccidiosis, changes in dietary formulation or feeding program, and environmental stressors (heat stress or high stocking density) disturb the intestinal microbiota and create a condition conducive to the overgrowth of *C. perfringens* [3]. The overgrowth of *C. perfringens* and the subsequent production of pore-forming toxins, such as alpha toxin (CPA) and necrotic enteritis b-like (NetB) toxin, can lead to damage of the small intestinal epithelial barrier, disruption of nutrient digestion and absorption, impaired growth performance, and significant mortality in severe cases [4,5]. Antibiotic growth promoters (AGPs) have been routinely used over decades to improve growth performance and control NE in chickens [6]. However, due to concerns over the emergence of antibiotic-resistant bacteria and the presence of antibiotic residues in animal production, many countries have implemented regulations to phase out the use of AGPs in animal feed [7]. This removal has resulted in impaired growth performance and an increased risk of enteric poultry diseases, such as NE [8]. The prevalence of *C. perfringens* has been reported to be higher in antibiotics-free flocks compared to conventional broiler flocks [9]. Therefore, several strategies, including biosecurity measures, vaccination, dietary intervention, and immunomodulators, have been employed to control NE in broiler chickens [10,11]. Modulating the innate immune system with immunomodulators can serve as an effective strategy to protect birds against microbial infections. Innate immunity is the first line of defense protecting the host against invading pathogens [12]. Recognition of conserved molecular motifs known as “microbe-associated molecular patterns (MAMPs) by pattern recognition receptors (PRRs) expressed by immune cells triggers downstream signaling pathways that lead to the activation of transcription factors (such as nuclear factor (NF)- kappaB) and the production of pro-inflammatory cytokines that orchestrate further immune response [13]. Bacterial DNA is among the MAMPs that have been found to stimulate the innate immune system. Toll-like receptor (TLR)-21, an avian PRR believed to function similarly to mammalian TLR9, can recognize synthetic and microbial unmethylated CpG oligodeoxynucleotides [14]. CpG oligonucleotides (ODN) are taken up by innate immune cells through endocytosis. Once internalized, they are delivered to endosomes where TLR21 is located [15]. This recognition triggers the activation of the innate immune system and mediates the host’s response to pathogens. Previous studies have reported the immunomodulatory and protective effects of synthetic CpG-oligodeoxynucleotides against various enteric pathogens in chickens. For example, Gomis and colleagues (2003) demonstrated that administering CpG-ODN (3.16 µg) via either the subcutaneous or intramuscular (IM) route, three days before the challenge, provides a protective effect against *Escherichia coli* in chickens [16]. Another study also demonstrated that IM administration of CpG-ODN (50 µg) on days 3 and 6 of age protects neonatal broiler chickens against *Salmonella* Typhimurium [17]. Abdelaziz and colleagues found that oral administration of encapsulated CpG-ODN (5 or 50 µg) 24 hours before challenge, reduces *Campylobacter* load in broilers [18]. Raj and colleagues demonstrated that the pretreatment of chickens with the CpG-ODN (10 or 50 µg) via the IM route enhances the expression of inflammatory cytokines, reduces oral and cloacal shedding of low pathogenic H9N2 avian influenza virus (AIV), and prevents fecal contact transmission of the virus in chickens [19,20]. Given the broad immunostimulatory activities of CpG-ODN, this study investigated its potential role in mitigating NE in broiler chickens.

## Materials and methods

### Animal housing

One-day-old male broiler chickens (n = 110) were obtained from a commercial hatchery (Curtis Chicks, a division of Maple Lodge hatcheries, Guelph, CA), weighed, and randomly assigned to five treatment groups. Birds were housed in separate floor pens with wood shaving and ad libitum access to feed and water in the isolation facility of the Ontario Veterinary College, University of Guelph. All animal experiments were approved by the Animal Care Committee of the University of Guelph and adhered to the guidelines of the Canadian Council on Animal Care (Animal Utilization Protocol # 4815).

### TLR ligand

The synthetic class B CpG ODN 2007 was obtained from Millipore-Sigma (Oakville, Ontario, Canada) and re-suspended in phosphate-buffered saline according to the manufacturer’s guidelines.

### NE challenge model

NE was experimentally induced in chickens as previously described [21]. The *C. perfringens* strain used in the present study was provided by Dr. John Prescott (University of Guelph). The bacteria were grown anaerobically at 37 °C overnight in Cooked Meat Medium (Thermo Fisher Scientific, Mississauga, ON, Canada). The overnight culture was then added to Fluid Thioglycollate medium (FTG; Millipore-Sigma, Oakville, ON, Canada) at 3% and incubated at 37 °C anaerobically for 15 h. The inoculum was used to challenge the birds.

### Experimental design

On day 21 of age, chickens (n = 110) were randomly assigned into five treatment groups: the first group (G1) was injected intramuscularly with 50 μg of CpG ODN in 100 µl phosphate-buffered saline (PBS); the second group (G2) was injected with 100 μg CpG IM; the third group (G3) was injected with 50 μg CpG followed by a booster dose on day 22; and the fourth (G4; positive control) and fifth (G5; negative control) groups were injected with a saline solution. Birds in G1-4 were orally challenged with *C. perfringens* (10^^8^ CFU/ml) twice daily for three days, starting on day 22 (day 22 to day 24 of age). On days 22 (18 hours after CpG injection and before *C. perfringens* challenge) and day 25 (termination date) tissue samples from the jejunum and ileum (six birds/group) were collected for gene expression analysis. On day 25, all birds were euthanized with CO2 inhalation, and intestinal lesions were scored (10 birds per group) as previously described [22]. Tissue samples from the jejunum and ileum were collected for flow cytometry and gut histomorphological analyses, and cecal contents (six birds per group) were collected for microbiome analysis. The experimental design is outlined in Table 1.

**Table 1:** Experimental groups.

Group	Treatment	CpG injection, d 21	CpG injection, d 22	C. perfringens challenge at days 22-24
**G1**	CpG, 50 μg	+	–	+
**G2**	CpG, 2 × 50 μg	+	+	+
**G3**	CpG, 100 μg	+	–	+
**G4**	Positive control (PC)	–	–	+
**G5**	Negative control (NC)	–	–	–

Chickens (n = 110) were randomly assigned to five treatment groups. On day 21 of age, chickens in groups G1-3 were injected intramuscularly with either 50 or 100 μg of CpG, and one group received a 50 μg dose followed by a booster on day 22. Birds in groups G1-4 were orally infected with *C. perfringens* twice daily from days 22 to 24.

### Histomorphometry

On day 25, 3 cm segments of the jejunum and ileum were collected, placed in tissue embedding cassettes, and preserved in a 10% formaldehyde solution for further processing. The tissues were then embedded, sectioned at 5 μm thickness, mounted on slides, and stained with Alcian blue (pH 2.5). The histology slides were imaged using an Olympus BX45 light microscope at 4X magnification. Villus height and crypt depth were measured using ImageJ software, and the villus height/crypt depth ratio was calculated.

### RNA extraction and reverse transcription

Tissue samples from jejunum and ileum segments were preserved in RNAlater before being homogenized with the Elite Bead Ruptor (Omni International, Kennesaw, GA, USA) using glass beads and 1 mL TRIzol® (Thermo Fisher Scientific, Mississauga, ON, Canada). RNA extraction followed the protocol outlined as described previously [23]. In brief, 200 µL of chloroform (Millipore-Sigma, Oakville, ON, Canada) was added to each homogenized sample and then incubated at room temperature for 15 minutes. The samples were centrifuged at 12,000 × g to separate the aqueous RNA phase, and the supernatant was transferred to a new tube. This phase separation was repeated, and 500 µL of ice-cold isopropanol was added to the supernatant. The samples were stored overnight at −20 °C to precipitate the RNA. After a subsequent centrifugation at 12,000 × g for 30 minutes, the RNA pellet was washed twice with ice-cold 75% ethanol and then incubated at 55°C for 10 minutes. RNA concentrations were measured using the NanoDrop™ 2000 spectrophotometer (ThermoFisher Scientific, Mississauga, ON, Canada) and adjusted to 10 µg before DNase treatment. After DNase treatment, RNA concentrations were measured again and adjusted to 1 µg/µL for complementary DNA (cDNA) synthesis. cDNA synthesis was performed using 500 ng of RNA and Oligo (dt) primers with the SuperScript First Strand System, according to the manufacturer’s instructions (Life Technologies, Canada). The resulting cDNA was diluted 1:10 in nuclease-free water and used as a template for real-time PCR.

### Real-time reverse transcription polymerase chain reaction

qRT-PCR was conducted using the LightCycler® 480 II system (Roche Diagnostics GmbH, Mannheim, DE) following the method described previously [23]. Each reaction included 10 μl of SYBR Green I master mix (Roche Diagnostics), 3 μL of PCR-grade water, 1 μL of forward primer, 1 μL of reverse primer, and 5 μLof diluted cDNA template. The PCR cycling protocol began with an initial denaturation at 95°C for 5 minutes, followed by 40-50 cycles of amplification with denaturation at 95°C for 20 seconds, annealing at 55°C–64°C (Table 2) for 10 seconds, and extension at 72°C for 10 seconds. Primers used in this study were synthesized by Millipore-Sigma (Oakville, ON) and are detailed in Table 2. Gene expression levels for interferon (IFN)-γ, interleukin (IL)-2, CXCL8, IL-10, and TLR21 mRNA transcripts were normalized to the housekeeping gene β-actin using the LightCycler® 480 software (Roche Diagnostics). 

**Table 2:** Primer sequences used for real-time quantitative PCR^1^.

Gene^2^	Primer sequence^3^ (5’-3’)	Annealing temperature	GeneBank accession number
*TLR21*	F: CCTGCGCAAGTGTCCGCTCA R: GCCCCAGGTCCAGGAAGCAG	60	NM_001030558
*IFN-γ*	F: ACACTGACAAGTCAAAGCCGCACA R: AGTCGTTCATCGGGAGCTTGGC	60	X99774
CXCL8	F: CCAAGCACACCTCTCTTCCA R: GCAAGGTAGGACGCTGGTAA	60	NM_205498.2
*IL-2*	F: TGCAGTGTTACCTGGGAGAAGTGGT R: ACTTCCGGTGTGATTTAGACCCGT	60	NM_204153.2
*IL-10*	F: AGCAGATCAAGGAGACGTTC R: ATCAGCAGGTACTCCTCGAT	60	AJ621614
*β-Actin*	F: CAACACAGTGCTGTCTGGTGGTA R: ATCGTACTCCTGCTTGCTGATCC	58	X00182

^1^The listed oligonucleotides were used to analyze gene expression via real-time quantitative PCR.

^2^IFN = Interferon; IL=Interleukin.

^3^F = forward; R = reverse.

### Intestinal mucosal mononuclear cell preparation and flow cytometry analysis

Mononuclear cell were isolated form intestinal tissues as described previously [24,25]. Tissue samples from the ileum were collected from all treatment groups (n = 5 birds/group) and immediately chilled on ice in PBS with penicillin-streptomycin (1%). Each tissue was then sectioned into small pieces (0.5 cm²) and rinsed three times with PBS. To extract mononuclear cells from the tissue sections, they were incubated at 37°C in PBS containing collagenase type 1 (Millipore-Sigma, Canada) at a concentration of 80 units/ml for 20 minutes. After incubation, the tissues were filtered through 40-μm cell strainers (Fisher Scientific, Mississauga, ON, Canada) using the rubber end of a 10-mL syringe plunger. The resulting cells were layered (2:1) onto Histopaque-1077 (Millipore-Sigma, Oakville, ON) and centrifuged at 600 × g) for 20 minutes at 21°C to allow for density gradient separation. Mononuclear cells were collected at the interface and washed twice with RPMI medium (Invitrogen, Burlington, Ontario, Canada) supplemented with 10% fetal bovine serum (Millipore-Sigma, Canada) and 1% penicillin-streptomycin. Cell counting was conducted using a hemocytometer and trypan blue exclusion method, after which cells were resuspended in RPMI medium at a density of 5 x 10^^6^/mL in 96-well plates. Following two washes in fluorescent activated cell sorting (FACS) buffer (PBS with 1% bovine serum albumin), cells were stained in the dark at 4°C for 30 minutes with fluorescent monoclonal antibodies. Two distinct surface staining panels were employed in this study. Panel 1 included mouse anti-chicken CD3-PB, mouse anti-chicken CD4-PE-Cy7, mouse anti-chicken CD8-APC, mouse anti-chicken CD25-FITC, and mouse anti-chicken TCRγδ-PE (TCR-1). Panel 2 comprised mouse anti-chicken Bu-1-PB, mouse anti-chicken IgM-APC, mouse anti-chicken monocyte/macrophage-FITC (KUL01), and mouse anti-major histocompatibility complex (MHC) II-PE. Antibodies were obtained from SouthernBiotech (SouthernBiotech, Birmingham, AL, USA). For the exclusion of dead cells in both staining panels, the fixable Live/Dead near-IR fluorescent reactive dye (Thermo Fisher Scientific, Canada) was employed. Appropriate controls were implemented to address non-specific binding and ensure proper compensation. Compensation was achieved using single-color stained controls, allowing precise adjustment for spectral overlaps between fluorochromes. Following staining, cells were washed twice in FACS buffer and fixed with 2% paraformaldehyde. Fixed cells were analyzed using a FACS Canto II flow cytometer (BD Bioscience, San Jose, CA, USA), equipped with the following lasers: 405 nm (violet), 488 nm (blue), 633 n (red) which enabled the detection of a broad range of fluorochromes. Data analysis was conducted using FlowJo software (v.10).

### DNA extraction and library preparation

On day 25 of age, the cereal contents were collected from six birds per treatment group and stored at -80°C. The genomic DNA of the cecal microbes was extracted using the QIAamp® Fast DNA Stool Mini Kit (Qiagen, Canada), following the method outlined by Alizadeh and colleagues [24]. The extracted DNA samples were sent to the Integrated Microbiome Resource (IMR) at Dalhousie University in Halifax, Nova Scotia, for library preparation and sequencing. To assess the composition of the cecal microbial communities, an Illumina 16S metagenomics sequencing workflow (Illumina Canada, Vancouver, BC, Canada) targeting the V3-V4 region of the 16S rRNA gene was employed, following the method described previously [26].

### Statistical analysis

Graphs were created, and statistical analyses were conducted using GraphPad Prism (Version 10). Data were analyzed with one-way ANOVA followed by Tukey’s multiple comparison test. When error deviations showed non-homogenous variance across treatment groups, a log transformation was applied. The normality of the data was assessed using the Shapiro–Wilk test. For data that did not follow a normal distribution, the Kruskal–Wallis nonparametric test was used, followed by Dunn’s test for multiple comparisons. Downstream processing of the 16S rRNA gene sequencing data was carried out using QIIME2 (2023.7), an integrated bioinformatics pipeline, following the protocols outlined in the Microbiome Helper pipeline [27]. Visualization was performed using the MicrobiomeAnalyst 2.0 web-based platform [28]. Individual alpha diversity for all samples was assessed using rarefaction curves, with the observed operational taxonomic unit (OTU) as the metric. Alpha diversity comparisons were made using the Shannon index. Beta diversity was illustrated using Principal Coordinates Analysis (PCoA) based on the weighted UniFrac distance matrix. The relative abundance at different taxonomic levels was displayed using stacked bar charts. Differential microbiota abundance analysis was conducted using Linear Discriminant Analysis Effect Size (LEfSe), which employs the Kruskal-Wallis rank sum test to identify features with significant differential abundance according to class labels, followed by Linear Discriminant Analysis to determine the relevance or effect size of the differentially abundant features. The corrected P value was set at P <  0.05.

## Results

### Intestinal lesion scores

**Fig 1:**
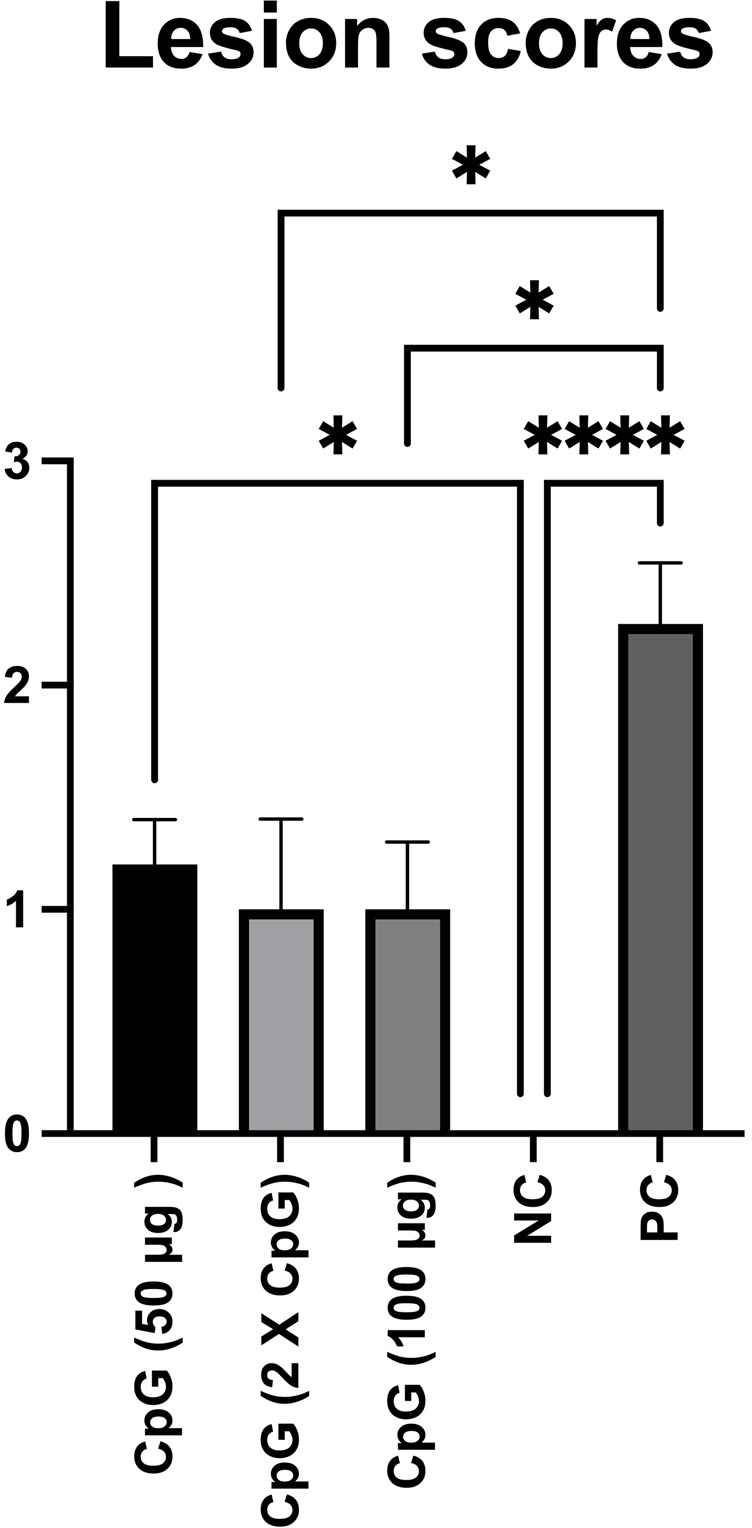
Intestinal lesion scores. Data represent mean gross lesion scores in the intestines of birds. On day 21 of age, chickens (n = 110) were randomly assigned to five treatment groups: the first group (G1) was injected with 50 μg CpG intramuscularly (IM); the second group (G2) was injected with 100 μg CpG IM; the third group (G3) was injected with 50 μg CpG followed by a booster dose on day 22; and the fourth (G4; positive control) and fifth (G5; negative control) groups were injected with a saline solution. Birds in G1-4 were orally challenged with *C. perfringens* from days 22 to 24. On day 25, 10 birds per group were euthanized, and lesion scoring was performed. Error bars represent the standard error of the mean. **P** values less than 0.05 are shown with one asterisk and **P** values less than 0.0001 are shown with four asterisks.

The results of gross pathology (Fig 1) showed that the mean lesion score in birds pretreated with CpG (100 μg or two doses of 50 μg) was lower than in the positive control group (untreated, *C. perfringens*-challenged).

### Expression of TLR21 and cytokines mRNA transcripts in the intestine

#### IL-2.

**Fig 2:**
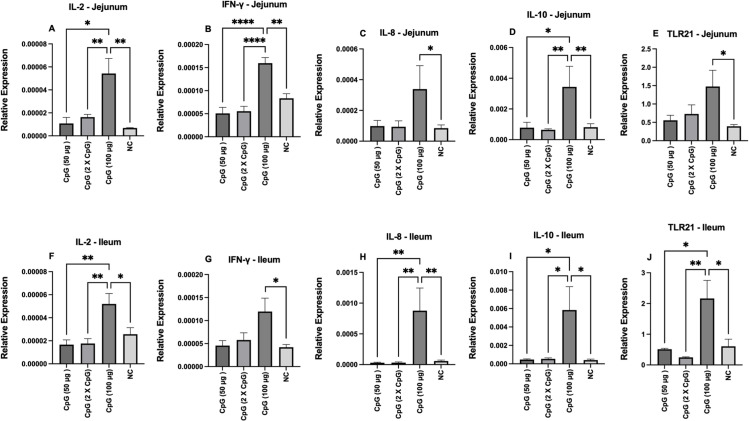
Gene expression of cytokines (IL-2, IFN-γ, IL8, and IL-10) and TLR21 at day 22 of age. Data represents the relative expression of IL-2 (A and F), IFN-γ (B and G), IL-8 (C and H), IL-10 (D and I), and TLR21 (E and J) in jejunum and ileum on day 22. On day 21 of age, chickens (n = 110) were randomly assigned to five treatment groups: the first group (G1) was injected with 50 μg CpG IM; the second group (G2) was injected with 100 μg CpG IM; the third group (G3) was injected with 50 μg CpG followed by a booster dose on day 22; and the fourth (G4; positive control) and fifth (G5; negative control) groups were injected with a saline solution. Birds in G1-4 were orally challenged with *C. perfringens* from days 22 to 24. On day 22 (18 hours after CpG injection), tissue samples from the jejunum and ileum (six birds per group) were collected for gene expression analysis. Error bars represent the standard error of the mean. **P** values less than 0.05 are shown with one asterisk**, P** values less than 0.01 are shown with two asterisks, **P** values less than 0.001 are shown with three asterisks, and **P** values less than 0.0001 are shown with four asterisks.

**Fig 3:**
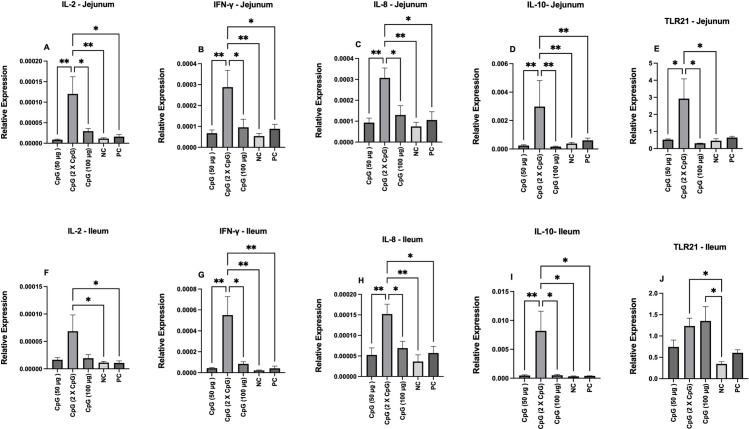
Gene expression of cytokines (IL-2, IFN-γ, IL8, and IL-10) and TLR21 at day 25 of age. Data represents the relative expression of IL-2 (A and F), IFN-γ (B and G), IL-8 (C and H), IL-10 (D and I), and TLR21 (E and J) in jejunum and ileum on day 25. On day 21 of age, chickens (n = 110) were randomly assigned to five treatment groups: the first group (G1) was injected with 50 μg CpG intramuscularly (IM); the second group (G2) was injected with 100 μg CpG IM; the third group (G3) was injected with 50 μg CpG followed by a booster dose on day 22; and the fourth (G4; positive control) and fifth (G5; negative control) groups were injected with a saline solution. Birds in G1-4 were orally challenged with *C. perfringens* from days 22 to 24. On day 25 (termination date), tissue samples from the Jejunum and ileum (six birds per group) were collected for gene expression analysis. Error bars represent the standard error of the mean. **P** values less than 0.05 are shown with one asterisk, and **P** values less than 0.01 are shown with two asterisks.

At day 22 of age, the relative expression of IL-2 mRNA transcripts was upregulated in the group pretreated with 100 μg of CpG compared to the other groups in both the jejunum and ileum (Fig 2A and F). At day 25 of age, the group pretreated twice with 50 μg of CpG exhibited higher IL-2 expression compared to the negative and positive control groups in both the jejunum and ileum (Fig 3A and F).

#### IFN-γ.

At day 22 of age, pretreatment of birds with 100 μg of CpG increased the relative expression of IFN-γ mRNA transcripts compared to the control group in the ileum and to the other groups in the jejunum (Fig 2B and G). At day 25 of age, pretreatment of birds twice with 50 μg of CpG enhanced IFN-γ expression compared to the other groups in both the jejunum and ileum (Fig 3B and G).

#### CXCL8.

At day 22 of age, birds pretreated with 100 μg of CpG showed higher expression of CXCL8 compared to the control group in the jejunum and to all other groups in the ileum (Fig 2C and H). At day 25 of age, the group pretreated twice with 50 μg of CpG exhibited higher expression of CXCL8 compared to the other groups in both the jejunum and ileum (Fig 3C and H).

#### IL-10.

At day 22 of age, pretreatment of birds with 100 μg of CpG increased the expression of IL-10 compared to other groups in both the jejunum and ileum (Fig 2D and I). At day 25 of age, pretreatment of birds twice with 50 μg of CpG increased IL-10 expression compared to the other groups in both the jejunum and ileum (Fig 3D and I).

#### TLR21.

At day 22 of age, birds pretreated with 100 μg of CpG showed higher expression of TLR21 compared to the control group in the jejunum and to all other groups in the ileum (Fig 2E and J). At day 25 of age, pretreatment of birds twice with 50 μg of CpG enhanced TLR21 expression in the jejunum compared to all groups except the positive control group. In addition, groups pretreated with 100 μg of CpG or twice with 50 μg of CpG enhanced TLR21 expression in the ileum (Fig 3E and J).

### Monocyte/macrophage and lymphocyte populations in the ileum

**Fig 4:**
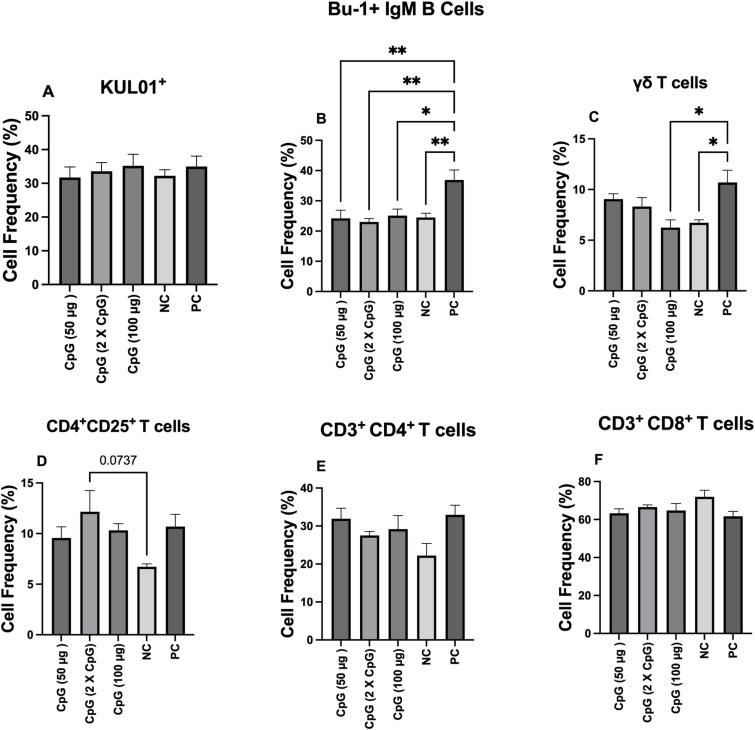
Frequency of monocyte/macrophages, Bu-1^+^ B cells, γδ T cells, CD3^+^CD4^+^ T cells, CD3^+^CD8^+^ T cells, and CD4^+^CD25^+^ T cells. Data represents the frequency of monocyte/macrophages (KUL01^+^; A), Bu-1^+^ B cells **(B)**, γδ T cells **(C)**, CD4^+^CD25^+^ T cells **(D)**, CD3^+^CD4^+^ T cells **(E)**, and CD3^+^CD8^+^ T cells (F) in the ileum on day 25. On day 21 of age, chickens (n = 110) were randomly assigned to five treatment groups: the first group (G1) was injected with 50 μg CpG IM; the second group (G2) was injected with 100 μg CpG IM; the third group (G3) was injected with 50 μg CpG followed by a booster dose on day 22; and the fourth (G4; positive control) and fifth (G5; negative control) groups were injected with a saline solution. Birds in G1-4 were orally challenged with *C. perfringens* from days 22 to 24. Tissue samples from the ileum (six birds per group) were collected on day 25 of age for flow cytometry analysis. Error bars represent the standard error of the mean. **P** values less than 0.05 are shown with one asterisk, and **P** values less than 0.01 are shown with two asterisks.

The results for cell populations in the ileum at day 25 of age showed that the frequency of monocytes/macrophages, CD3^ + ^CD4^ +^ T cells, and CD3^ + ^CD8^ +^ T cells were not affected by the pretreatment groups (Fig 4A, D, E and F). However, the positive control group showed a higher frequency of Bu-1^ +^ B cells compared to the other treatment groups (Fig 4B). Additionally, the positive control group increased the frequency of γδ T cells compared to the negative control group and the group pretreated with 100 μg CpG (Fig 4C and F).

### Gut histomorphology

**Fig 5:**
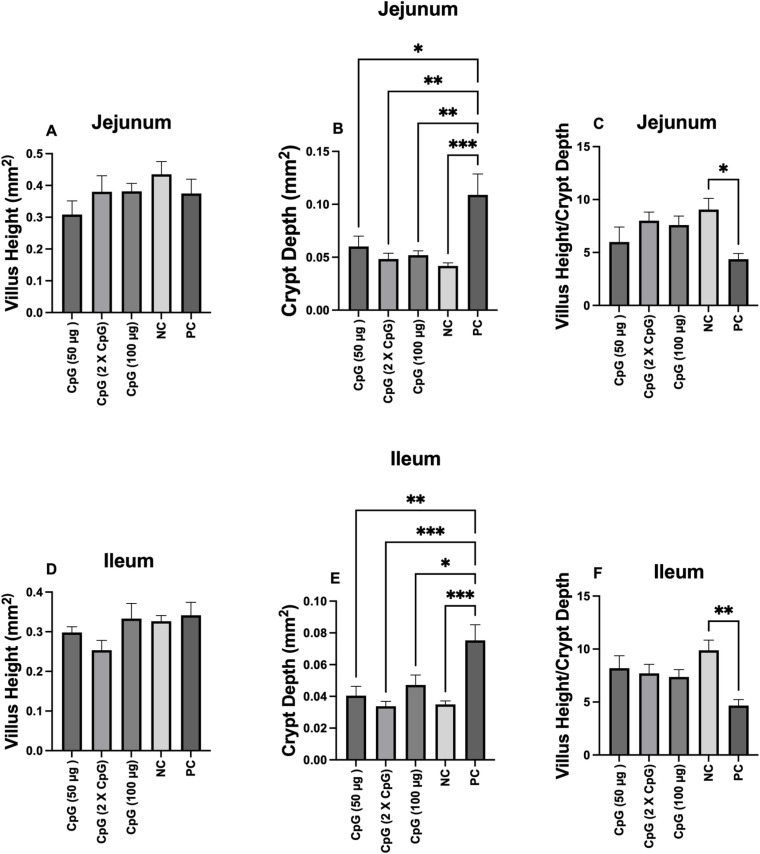
Villus height, crypt depth, and villus height/crypt depth ratio. Data represents villus height (A and D), crypt depth (B and E), and villus height\crypt depth ratio (C and F) in jejunum and ileum at day 25. On day 21 of age, chickens (n = 110) were randomly assigned to five treatment groups: the first group (G1) was injected with 50 μg CpG IM; the second group (G2) was injected with 100 μg CpG IM; the third group (G3) was injected with 50 μg CpG followed by a booster dose on day 22; and the fourth (G4; positive control) and fifth (G5; negative control) groups were injected with a saline solution. Birds in G1-4 were orally challenged with *C. perfringens* from days 22 to 24. On day 25 of age, birds were euthanized, and tissue segments from the jejunum and ileum (six birds per group) were collected for histomorphology analysis. Error bars represent the standard error of the mean. Results were considered statistically significant if ****P**** <  0.05. **P** values less than 0.05 are shown with one asterisk**, P** values less than 0.01 are shown with two asterisks, and **P** values less than 0.001 are shown with three asterisks.

The villus height in the jejunum and ileum was not affected by CpG pretreatment or *C. perfringens* challenge (Fig 5A and D). However, the positive control group increased crypt depth compared to the other groups (Fig 5B and E). In addition, the negative control group had a higher villus height to crypt depth (VH:CD) ratio compared to the positive control group (Fig 5C and F).

### Cecal bacterial community

**Fig 6:**
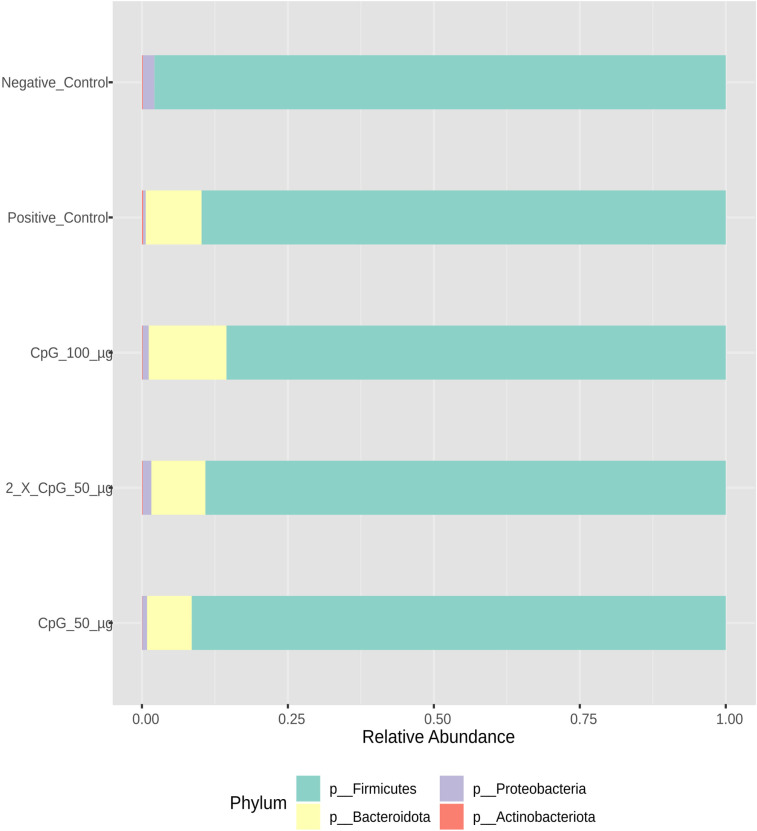
Taxonomic composition of cecal bacterial communities at phylum level. Data represents the taxonomic composition of broiler chicken cecal bacterial communities at the phylum level. On day 21 of age, chickens (n = 110) were randomly assigned to five treatment groups: the first group (G1) was injected with 50 μg CpG IM; the second group (G2) was injected with 100 μg CpG IM; the third group (G3) was injected with 50 μg CpG followed by a booster dose on day 22; and the fourth (G4; positive control) and fifth (G5; negative control) groups were injected with a saline solution. Birds in G1-4 were orally challenged with *C. perfringens* from days 22 to 24. On day 25, birds were euthanized, and cecal contents (six birds per group) were collected for microbiome analysis.

**Fig 7:**
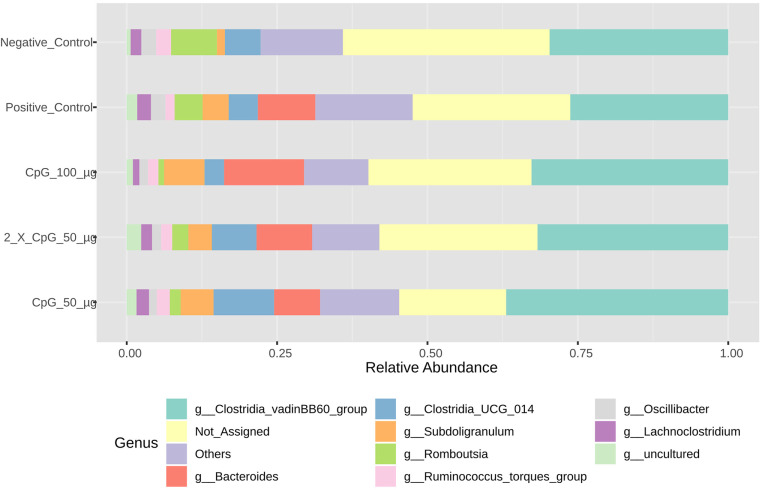
Taxonomic composition of cecal bacterial communities at the genus level. Data represents the taxonomic composition of broiler chicken cecal bacterial communities at the genus level. On day 21 of age, chickens (n = 110) were randomly assigned to five treatment groups: the first group (G1) was injected with 50 μg CpG IM; the second group (G2) was injected with 100 μg CpG IM; the third group (G3) was injected with 50 μg CpG followed by a booster dose on day 22; and the fourth (G4; positive control) and fifth (G5; negative control) groups were injected with a saline solution. Birds in G1-4 were orally challenged with *C. perfringens* from days 22 to 24. On day 25, birds were euthanized, and cecal contents (six birds per group) were collected for microbiome analysis.

Profiling the bacterial composition of collected cecal samples on day 25 of age revealed that Firmicutes (>80%), Bacteroidota ( ≈ 12.5%), and Proteobacteria ( ≈ 2.5%) were the predominant phyla (Fig 6). At the genus level, *Clostridia*, *Subdoligranulum*, *Romboutsia*, *Ruminococcus torques* group, *Oscillibacter,* and *Lachnoclostridium* were the most abundant (Fig 7). A shift in bacterial composition driven by the *C. perfringens* challenge was also observed. Specifically, the genus Bacteroides was present in all treatment groups except the negative control group. Additionally, higher abundances of the genera *Romboutsia* and *Ruminococcus torques* group were found in the negative control group compared to other treatment groups. Conversely, a higher abundance of the genus *Subdoligranulum* was observed in all *C. perfringens* -challenged treatment groups relative to the negative control group. The relative abundance profiles of other taxonomic groups are presented in S1–S3 Figs. Rarefaction curves of species richness were used to estimate internal sample alpha diversity. The results revealed that sequencing depth was adequate to cover the bacterial diversity in the cecal samples (S4 Fig).

### Alpha diversity of the cecal microbiome

**Fig 8:**
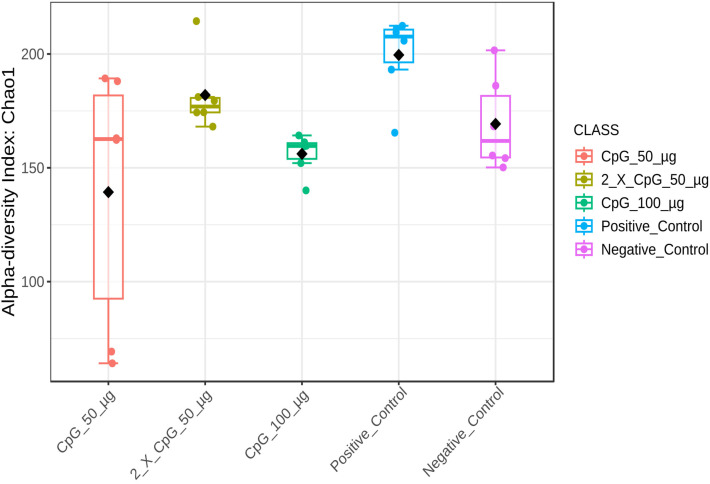
Alpha diversity of cecal microbiome - Chao1 index. Alpha diversity metric, Chao1 index,) of the cecal bacterial community in broiler chickens as affected by treatments. Boxplots display interquartile ranges, with medians represented by solid lines, means by diamond shapes within the boxes, and outliers shown outside the boxes. The treatment groups were as follows: the first group (G1) was injected with 50 μg CpG IM; the second group (G2) was injected with 100 μg CpG IM; the third group (G3) was injected with 50 μg CpG, followed by a booster dose on day 22; and the fourth (G4; positive control) and fifth (G5; negative control) groups were injected with a saline solution. Birds in G1-4 were orally challenged with *C. perfringens* from days 22 to 24. On day 25, birds were euthanized, and cecal contents (six birds per group) were collected for microbiome analysis.

**Fig 9:**
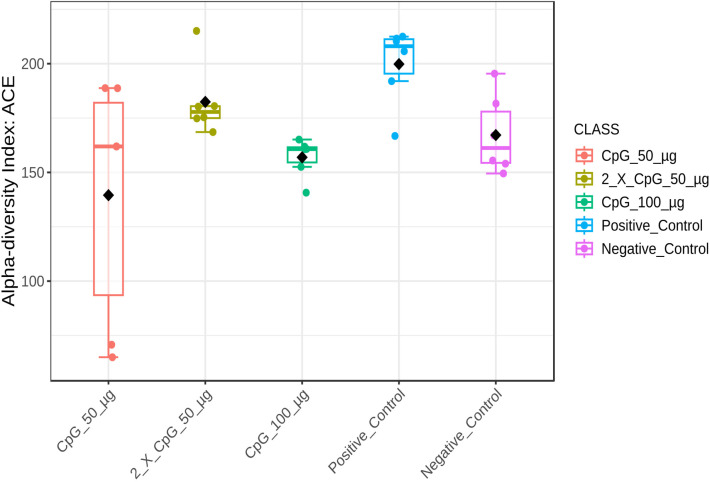
Alpha diversity of cecal microbiome - Ace index. Alpha diversity metric, Ace index, of the cecal bacterial community in broiler chickens as affected by treatments. Boxplots display interquartile ranges, with medians represented by solid lines, means by diamond shapes within the boxes, and outliers shown outside the boxes. The treatment groups were as follows: the first group (G1) was injected with 50 μg CpG IM; the second group (G2) was injected with 100 μg CpG IM; the third group (G3) was injected with 50 μg CpG, followed by a booster dose on day 22; and the fourth (G4; positive control) and fifth (G5; negative control) groups were injected with a saline solution. Birds in G1-4 were orally challenged with *C. perfringens* from days 22 to 24. On day 25, birds were euthanized, and cecal contents (six birds per group) were collected for microbiome analysis.

**Fig 10:**
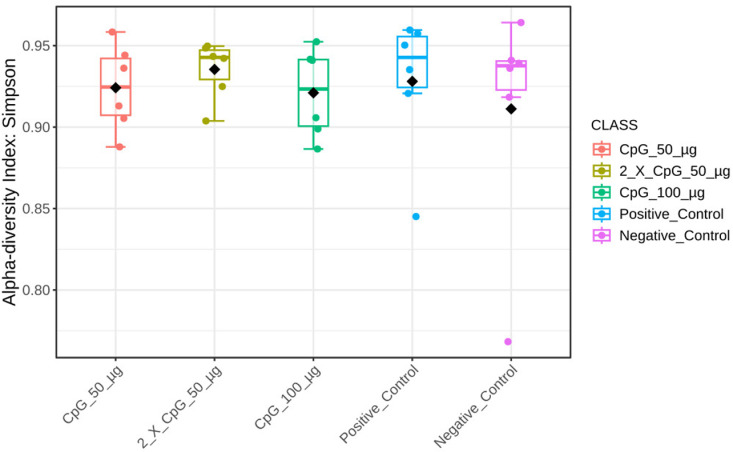
Alpha diversity of cecal microbiome – Simpson index. Alpha diversity metric, Simpson index, of the cecal bacterial community in broiler chickens as affected by treatments. Boxplots display interquartile ranges, with medians represented by solid lines, means by diamond shapes within the boxes, and outliers shown outside the boxes. The treatment groups were as follows: the first group (G1) was injected with 50 μg CpG IM; the second group (G2) was injected with 100 μg CpG IM; the third group (G3) was injected with 50 μg CpG, followed by a booster dose on day 22; and the fourth (G4; positive control) and fifth (G5; negative control) groups were injected with a saline solution. Birds in G1-4 were orally challenged with *C. perfringens* from days 22 to 24. On day 25, birds were euthanized, and cecal contents (six birds per group) were collected for microbiome analysis.

**Fig 11:**
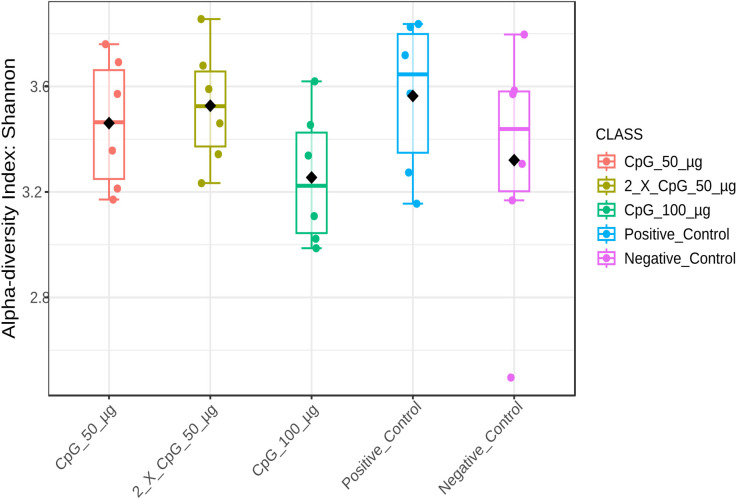
Alpha diversity of cecal microbiome – Shannon index. Alpha diversity metric, Shannon index, of the cecal bacterial community in broiler chickens as affected by treatments. Boxplots display interquartile ranges, with medians represented by solid lines, means by diamond shapes within the boxes, and outliers shown outside the boxes. The treatment groups were as follows: the first group (G1) was injected with 50 μg CpG IM; the second group (G2) was injected with 100 μg CpG IM; the third group (G3) was injected with 50 μg CpG, followed by a booster dose on day 22; and the fourth (G4; positive control) and fifth (G5; negative control) groups were injected with a saline solution. Birds in G1-4 were orally challenged with *C. perfringens* from days 22 to 24. On day 25, birds were euthanized, and cecal contents (six birds per group) were collected for microbiome analysis.

To comprehensively analyze and compare the cecal bacterial community structure in broiler chickens subjected to *C. perfringens* infection and various CpG pre-treatments, alpha-diversity metrics such as Chao1, Ace, Simpson, and Shannon indices were assessed (Figs 8–11). The results showed that diversity metrics measuring species richness (Chao1 and Ace) indicated the positive control treatment had higher species richness than all other treatments, except for the group that received the two doses of CpG 50 µg. Among the CpG pre-treatments, the group that received the two doses of CpG 50 µg exhibited higher species richness compared to the CpG 100 µg treatment (S1 and S2 Tables). However, no differences in alpha diversity were observed between the treatments when diversity metrics that account for both species richness and evenness (Simpson and Shannon indices) were calculated.

### Beta diversity of the cecal microbiome

**Fig 12:**
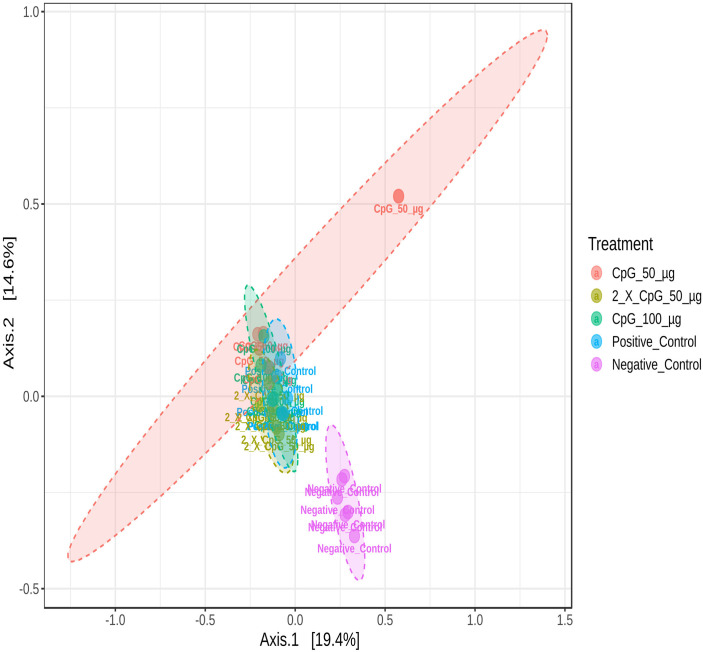
Beta diversity of cecal microbiome. PCoA plots based on the weighted UniFrac metric, illustrating variance in cecal microbial communities between treatment groups. The treatment groups were as follows: the first group (G1) was injected with 50 μg CpG IM; the second group (G2) was injected with 100 μg CpG IM; the third group (G3) was injected with 50 μg CpG, followed by a booster dose on day 22; and the fourth (G4; positive control) and fifth (G5; negative control) groups were injected with a saline solution. Birds in G1-4 were orally challenged with *C. perfringens* from days 22 to 24. On day 25, birds were euthanized, and cecal contents (six birds per group) were collected for microbiome analysis.

To further explore similarity and dissimilarity between groups or differences in composition among communities, a quantitative analysis of community profiles was performed using principal coordinate analysis (PCoA) based on Bray-Curtis distance matrices and the corresponding PERMANOVA statistics (Fig 12). *C. perfringens* challenge was observed to explain the variance in the chicken gut microbial community between treatment groups. Unique clustering was observed in the CpG-pretreated groups and the positive control group compared to the unchallenged group suggesting that *C. perfringens* infection results in significant changes in cecal microbiota diversity.

### Abundance of individual bacterial taxa in the cecum

**Fig 13:**
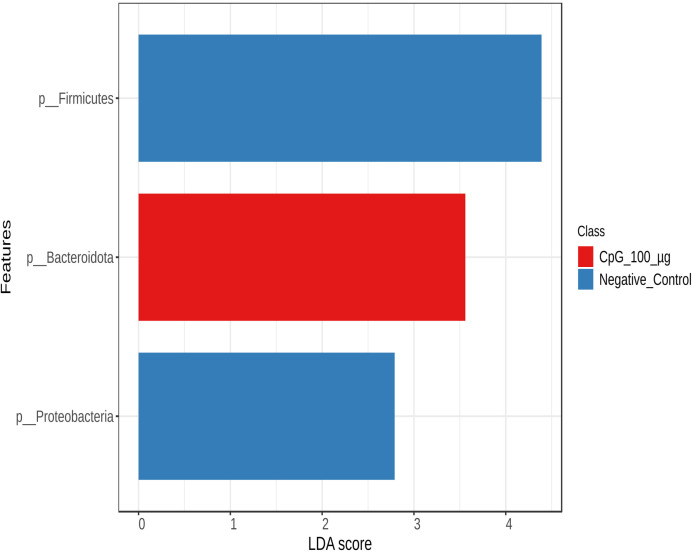
Abundance of bacterial taxa at the phylum level. Data represents the changes observed in the abundance of bacterial taxa at the phylum level.On day 21 of age, chickens (n = 110) were randomly assigned to five treatment groups: the first group (G1) was injected with 50 μg CpG IM; the second group (G2) was injected with 100 μg CpG IM; the third group (G3) was injected with 50 μg CpG, followed by a booster dose on day 22; and the fourth (G4; positive control) and fifth (G5; negative control) groups were injected with a saline solution. Birds in G1-4 were orally challenged with *C. perfringens* from days 22 to 24. On day 25, birds were euthanized, and cecal contents (six birds per group) were collected for microbiome analysis. Horizontal bars represent the log fold change ±  standard error (depicted as error bars) in absolute abundance associated with each treatment, as determined by Linear Discriminant Analysis Effect Size (LEfSe). Only taxa exhibiting statistically significant changes in abundance are presented.

**Fig 14:**
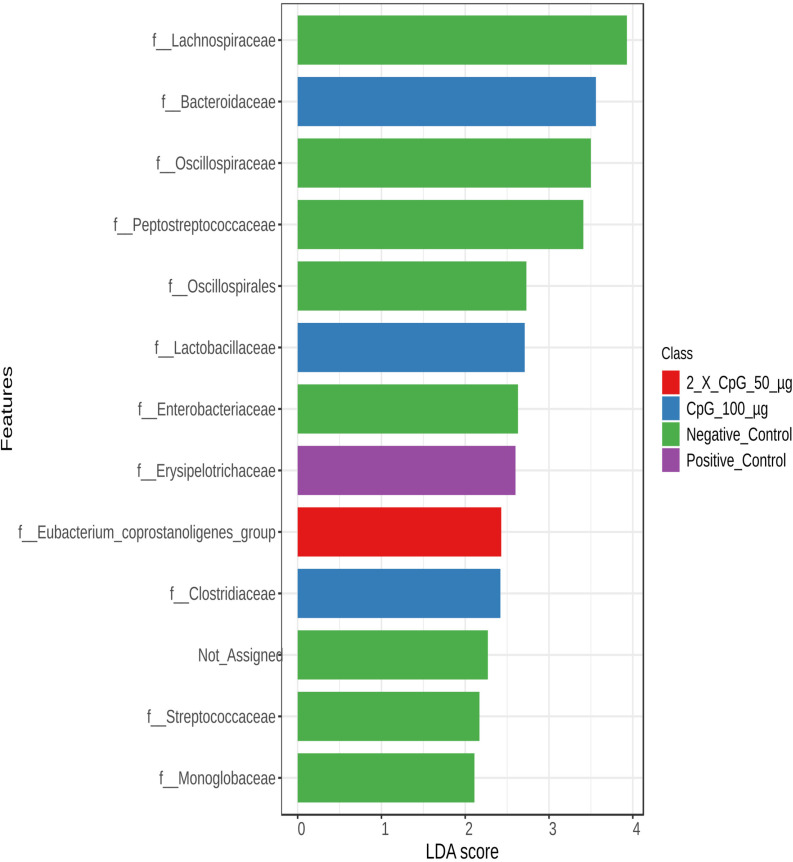
Abundance of bacterial taxa at the family level. Data represents the changes observed in the abundance of bacterial taxa at the family level. On day 21 of age, chickens (n = 110) were randomly assigned to five treatment groups: the first group (G1) was injected with 50 μg CpG IM; the second group (G2) was injected with 100 μg CpG IM; the third group (G3) was injected with 50 μg CpG, followed by a booster dose on day 22; and the fourth (G4; positive control) and fifth (G5; negative control) groups were injected with a saline solution. Birds in G1-4 were orally challenged with *C. perfringens* from days 22 to 24. On day 25, birds were euthanized, and cecal contents (six birds per group) were collected for microbiome analysis. Horizontal bars represent the log fold change ±  standard error (depicted as error bars) in absolute abundance associated with each treatment, as determined by Linear Discriminant Analysis Effect Size (LEfSe). Only taxa exhibiting statistically significant changes in abundance are presented.

**Fig 15:**
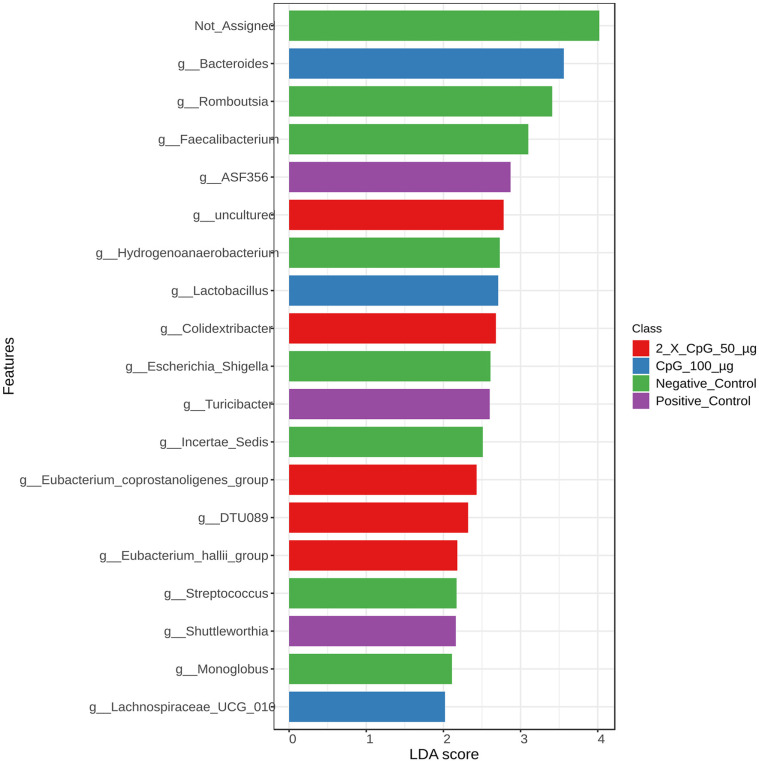
Abundance of bacterial taxa at the genus level. Data represents the changes observed in the abundance of bacterial taxa at the genus level. On day 21 of age, chickens (n = 110) were randomly assigned to five treatment groups: the first group (G1) was injected with 50 μg CpG IM; the second group (G2) was injected with 100 μg CpG IM; the third group (G3) was injected with 50 μg CpG, followed by a booster dose on day 22; and the fourth (G4; positive control) and fifth (G5; negative control) groups were injected with a saline solution. Birds in G1-4 were orally challenged with *C. perfringens* from days 22 to 24. On day 25, birds were euthanized, and cecal contents (six birds per group) were collected for microbiome analysis. Horizontal bars represent the log fold change ±  standard error (depicted as error bars) in absolute abundance associated with each treatment, as determined by Linear Discriminant Analysis Effect Size (LEfSe). Only taxa exhibiting statistically significant changes in abundance are presented.

**Fig 16:**
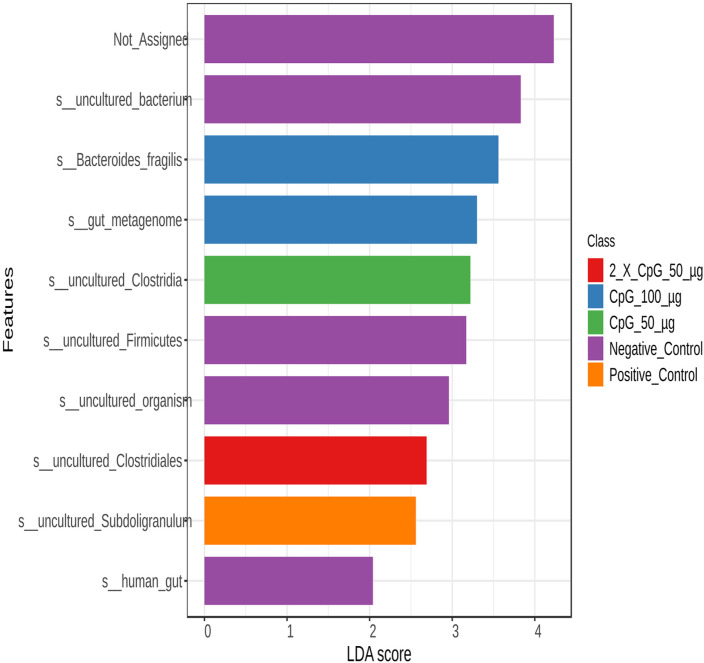
Abundance of bacterial taxa at the species level. Data represents the changes observed in the abundance of bacterial taxa at species level. On day 21 of age, chickens (n = 110) were randomly assigned to five treatment groups: the first group (G1) was injected with 50 μg CpG IM; the second group (G2) was injected with 100 μg CpG IM; the third group (G3) was injected with 50 μg CpG, followed by a booster dose on day 22; and the fourth (G4; positive control) and fifth (G5; negative control) groups were injected with a saline solution. Birds in G1-4 were orally challenged with *C. perfringens* from days 22 to 24. On day 25, birds were euthanized, and cecal contents (six birds per group) were collected for microbiome analysis. Horizontal bars represent the log fold change ±  standard error (depicted as error bars) in absolute abundance associated with each treatment, as determined by Linear Discriminant Analysis Effect Size (LEfSe). Only taxa exhibiting statistically significant changes in abundance are presented.

To examine variations in bacterial community structures across the treatment groups, LEfSe was used to perform an abundance analysis and identify key bacterial taxa, the absolute abundance of which was significantly affected by the specific treatments (Figs 13–16).

At the phylum level (Fig 13), birds in the unchallenged group (negative control) exhibited a differential abundance of bacteria belonging to the phyla Firmicutes and Proteobacteria, while the group pretreated with 100 µg showed greater abundance of Bacteroidota.

At the family level (Fig 14), the positive control group exhibited a high abundance of bacteria in the family *Erysipelotrichaceae*, while a high abundance of bacteria in the families *Monoglobaceae*, *Streptococcaceae*, *Clostridiaceae*, *Eubacterium coprostanoligenes* group, *Enterobacteriaceae*, *Oscillospirales*, *Peptostreptococcaceae*, *Oscillospiracea*e, and *Lachnospiraceae*, was observed in the negative control group. Pretreatment of birds with the 100 µg of CpG recorded a higher abundance of bacteria in the families *Lactobacillaceae*, *Bacteroidaceae*. and *Colstridaceae*. Moreover, the family of *Eubacterium-Coprostanoligenes* was more abundant in the group that received the two doses of 50 µg CpG pre-treatment.

At the genus level (Fig 15), signature genera associated with the unchallenged group (negative control) included *Monoglobus, Streptococcus, Incertae Sedis*, *Escherichia_Shigella*, *Hydrogennaerobacterium*, *Faecalibacterium*, and *Romboutsia*. Signature genera associated with the positive control treatment included *Shuttleworthia*, *Tunicibacter*, and unclassified ASF356. Pretreatment of birds with 100 µg of CpG increased the abundance of the genera *Bacteroides*, *Lactobacillus* and *Lachnospiraceae_UCG-010*. Additionally, the group that was pretreated with the two doses of 50 µg CpG was enriched with genera *DTU089*, *Eubacterium-hallii group*, and *Eubacterium-Coprostanoligenes group*.

At the species level (Fig 16), the positive control group was differentially enriched with the uncultured *Subdoligranulum*, while the unchallenged group (negative control) was differentially enriched with the human gut-associated species and uncultured Firmicutes. Pretreatment of birds with 50 µg CpG resulted in a higher abundance of uncultured *Clostridia* species, while the 100 µg CpG dose resulted in a higher abundance of species belonging to *gut metagenome* and *Bacteroides fragilis*. The group that received two doses of 50 µg CpG showed a higher abundance of uncultured *Clostridiales* species.

## Discussion

NE is one of the most important enteric diseases of poultry, traditionally controlled by AGPs [4]. However, the use of AGPs has been limited due to regulatory measures and concerns over antibiotic resistance. Therefore, alternative strategies have been implemented to control NE in broiler chickens [10]. Immunomodulation of chickens is one strategy that can increase the natural defenses of chickens against a variety of pathogens [29]. Therefore, the present study evaluated the effects of CpG, a TLR ligand and an immunomodulator in chickens against *C. perfringens*.

Overall, the results of this study demonstrated that pretreatment of birds with intramuscular administration of CpG ODN induced the expression of TLR21 and cytokines mRNA transcripts, altered the gut microbiome composition, and was associated with a reduction in gross lesion scores in the intestines following a *C. perfringens* challenge. However, it should be noted that the observed effects are largely dependent on the concentration of CpG and/or the frequency of administration. For instance, while administration of CpG at a dose of 100 μg or two doses of 50 μg reduced the mean lesion score in the intestine compared to the positive control group, no such effects were observed in the group that received the lower dose of CpG (50 μg).

The intestinal tract, the largest immune organ in chickens, contains various lymphoid organs, including Peyer’s patches, Meckel’s diverticulum, cecal tonsils, and other lymphoid aggregates. It also houses a diverse array of cells with immune functions, such as epithelial cells, intraepithelial, and lamina propria lymphocytes [30]. These cells express various types of TLRs, including TLR21, that play a critical role in recognizing PAMPs [31]. TLR21 recognizes microbial unmethylated CpG DNA motifs and activates downstream signaling pathways, leading to the production of cytokines and chemokines that initiate various immune responses [14]. In the present study, the underlying immunological mechanisms of protection associated with the administration of CpG were investigated by measuring the expression levels of TLR21 and various cytokines in the jejunum and ileum. The findings, irrespective of CpG dosage, indicated elevated levels of IL-2, CXCL8, IL-10, and IFN-γ in both intestinal regions, suggesting that these cytokines may contribute to the observed protection against NE. Additionally, the simultaneous expression of pro-and anti-inflammatory cytokines highlights the role of CpG in maintaining immune homeostasis via negative feedback. These findings align with previous studies that have demonstrated a link between CpG-induced cytokine expression and enhanced protection against *E. coli*, *Salmonella*, and *Campylobacter* infections in chickens [16–18,32].

At the cellular level, CpG administration did not affect the frequency of monocytes/macrophages, αβ and γδ T cells, or B cells in the ileum of pretreated birds, indicating that CpG pretreatment may not impact lymphocyte infiltration and proliferation in the intestine. On the other hand, infection with *C. perfringens* in the positive control group led to an increase in the frequency of γδ T cells compared to both the negative control group and the group pretreated with 100 μg of CpG. γδ T cells are relatively abundant in the small intestine of chickens compared to other species and play a critical role in the early immune response to intestinal infections, maintaining intestinal epithelial integrity, and tissue repair during intestinal infection [33,34]. Evidence suggests that intestinal infections increase the number of γδ T cells at the site of infection through various mechanisms, including pathogen recognition, chemokine-mediated recruitment, and cytokine signaling [35]. Therefore, a higher number of γδ T cells in the ileum might indicate increased inflammation and intestinal damage, reflecting the host response to restore the affected tissue.

Similarly, B cells are also a key component of intestinal mucosal immunity and contribute to the regulation of immune responses [36]. Our study demonstrated that *C. perfringens* infection increased the frequency of Bu-1^ +^ B cells compared to CpG-pretreated and negative control groups. This increase suggests that intestinal infections can induce inflammation and damage to the intestinal epithelium, leading to B cell activation and proliferation [37]. Considering the role of B cells in regulating immune responses and maintaining intestinal immune homeostasis during infection [38] the lower number of B cells observed in the CpG-pretreated and negative control groups suggests a healthier intestine with less inflammation compared to the positive control group.

In the present study, the impact of CpG on maintaining gut integrity during *C. perfringens* infection was evaluated through morphometric analysis of the small intestine. While pre-treatment of birds with intramuscular administration of CpG did not affect villus height, the positive control group showed higher crypt depth compared to both the CpG-pretreated and negative control groups. This increased crypt depth in the positive control group may indicate enhanced cell proliferation. Crypt-base columnar cells, which are continuously dividing intestinal stem cells, play a crucial role in generating all differentiated epithelial cell types [39,40]. The disruption of epithelial cells by *C. perfringens* and its toxins can lead to increased cell turnover and regeneration as a compensatory mechanism to replace damaged epithelial cells, resulting in deeper crypts or crypt hyperplasia [41,42]. Conversely, lower crypt depth in the CpG-pretreated groups might indicate less intestinal damage and turnover, which was supported by reduced lesion scores in the intestine. Additionally, the increased VH:CD ratio in the negative control group compared to the positive control group is an important indicator of intestinal health and function. Damage to the epithelial cells of the villi by *C. perfringens* and its toxins may lead to villus atrophy and crypt hyperplasia, resulting in a lower VH:CD ratio. Previous studies by our group also demonstrated that challenging birds with *C. perfringens* can lead to crypt hyperplasia and a decrease in the VH:CD ratio [21,43]. Additionally, Wang and colleague also found that *C. perfringens*-induced NE led to crypt hyperplasia compared to uninfected birds in chickens [44].

The results of the cecal microbiota composition assessed on day 25 of age indicated that alpha diversity, measured by Simpson and Shannon diversity indices, was not affected by the treatment groups. However, beta diversity based on Bray-Curtis distance matrices indicated significant differences between challenged and unchallenged treatment groups. Additionally, alpha diversity measured by Chao1 and Ace indices revealed that the positive control treatment had higher species richness than all other treatments, except for the group that received CpG at two doses. Consistent with the observed results, several other poultry studies involving immunological challenges, including *C. perfringens* challenge, reported no changes in alpha diversity, as indicated by Shannon diversity index, but distinct differences in beta diversity [45–48]. This suggests that while *C. perfringens* did not alter species evenness in the cecum, it did induce significant alterations in community structure. The composition and diversity of poultry microbiota are influenced by various factors, including intrinsic host-related factors such as bird type, breed, age, and genetics, as well as extrinsic factors such as environmental stressors, nutrition, sampling methods, sites, and time [49]. Additionally, despite the temporal disturbance caused by *C. perfringens* challenge, the cecal lumen may possess inherent resilience and immune responses that allow microbial species to maintain dynamic equilibrium. A similar observation has been made in a mixed *Eimeria spp.* challenge involving broiler chickens [50].

Collectively, pretreatment of birds with intramuscular administration of 100 μg of CpG positively influenced the cecal microbiota composition in chickens challenged with *C.* perfringens. Pretreatment at this dose modality increased the proportion of the phylum *Bacteroidota*, the family *Bacteroidaceae*, and the genus *Bacteroides*, which play key roles in promoting gut health through immune regulation, gut barrier maintenance, and microbial balance [51]. These bacteria have been shown to inhibit the colonization of pathogenic microbes through competitive exclusion. They are also involved in the breakdown of complex carbohydrates and the production of short-chain fatty acids (SCFAs) [52,53]. SCFAs serve as an energy source for intestinal epithelial cells and possess anti-inflammatory properties [54]. Additionally, SCFAs contribute to immune system modulation and help maintain the integrity of the gut barrier by strengthening tight junctions between intestinal cells, reducing gut permeability, thereby preventing the translocation of pathogens and their toxins from the gut to the lamina propria [55].

At the species level, pretreatment of birds with an intramuscular dose of 100 μg CpG increased the proportion of *Bacteroides fragilis*, which belongs to the genus *Bacteroides*. *Bacteroides fragilis* is known for its role in immune system regulation by promoting T cell differentiation and enhancing the production of anti-inflammatory cytokines. It also contributes to maintaining the gut barrier by promoting mucus production and supporting a balanced microbial community through the production of SCFAs and antimicrobial compounds that suppress pathogenic bacteria [56]. Further, pretreatment with 100 μg of CpG increased the abundance of the family *Lactobacillaceae* and the genus *Lactobacillus. Lactobacillus*, a member of the lactic acid bacteria (LAB) group, is known for maintaining a healthy gut microbiota balance, immunomodulation, and preventing infectious diseases [57]. The increased abundance of *Lactobacillaceae* and *Lactobacillus* in birds pretreated with 100 μg of CpG suggests a healthier gut, as evidenced by a lower lesion score in the intestine.

## Conclusions

In conclusion, the results of the present study demonstrated that pretreatment with intramuscular administration of CpG ODN reduced mean lesion scores in the intestines of chickens in a dose-dependent manner following the *C. perfringens* challenge. Specifically, pretreatment of CpG ODN at a dose of 100 μg enhanced intestinal immune responses against NE infection by increasing the expression of TLR21 and various cytokines and maintaining intestinal morphology and gut microbiota during *C. perfringens* infection. These findings underscore the potential of CpG ODN as an immune-based approach to mitigate NE in broiler chickens.

## Supporting information

S1 Fig Taxonomic composition of dominant classes in cecal microbiota as affected by treatments. (DOCX)

S2 Fig Taxonomic composition of dominant orders in cecal microbiota as affected by treatments. (DOCX)

S3 Fig Taxonomic composition of dominant family in cecal microbiota as affected by treatments. (DOCX)

S4 Fig Rarefaction curves of species richness and samples obtained from 16S rRNA gene V3–V4 - Sequences based on treatment groups. (DOCX)

S1 Table Post-hoc comparisons of alpha diversity (Ace index) between treatment groups. (DOCX)

S2 Table Post-hoc comparisons of alpha diversity (Chao1 index) between treatment groups. (DOCX)
