# Supplementary material for: Evaluating the protective effects of the Toll-like receptor (TLR) 21 ligand, CpG ODN, against necrotic enteritis in broiler chickens
Source: PLoS One. 2025 Mar 13;20(3):e0319404. doi: 10.1371/journal.pone.0319404 (PMC11906054; doi:10.1371/journal.pone.0319404)
Supplement: S1 Fig — (DOCX) [file pone.0319404.s001.docx]

**Supplementary information 1**


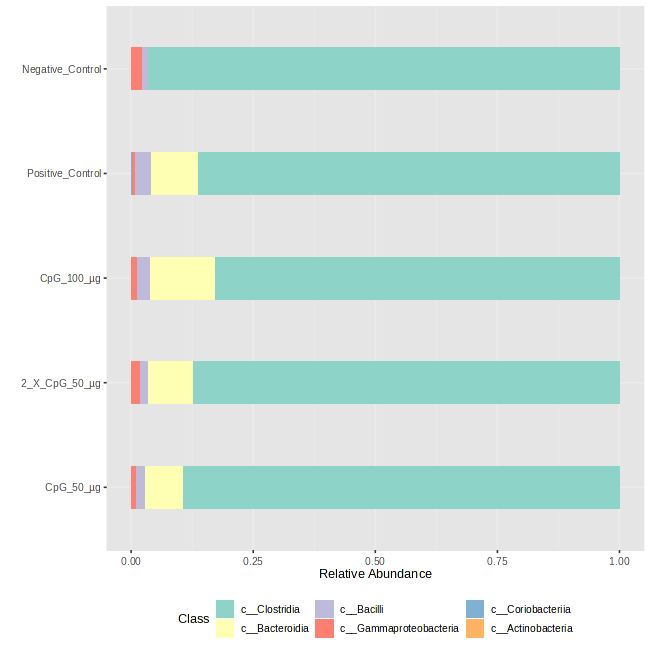


**Taxonomic composition of dominant classes in cecal microbiota as affected by treatments**: the first group (G1) was injected with 50 μg CpG intramuscularly (IM); the second group (G2) was injected with 100 μg CpG IM; the third group (G3) was injected with 50 μg CpG followed by a booster dose on day 22 post-hatch; and the fourth (G4; positive control) and fifth (G5; negative control) groups were injected with a saline solution. Birds in G1-4 were challenged on day 22 post-hatch with *C. perfringens*.
