# Supplementary material for: Evaluating the protective effects of the Toll-like receptor (TLR) 21 ligand, CpG ODN, against necrotic enteritis in broiler chickens
Source: PLoS One. 2025 Mar 13;20(3):e0319404. doi: 10.1371/journal.pone.0319404 (PMC11906054; doi:10.1371/journal.pone.0319404)
Supplement: S1 Table — (DOCX) [file pone.0319404.s005.docx]

**Supplementary information 5**

| Treatment | P-value | Statistical Significance |
| --- | --- | --- |
| Negative_Control vs 2X_CpG_50_ug | 0.1583 | NS |
| Negative_Control vs CpG_100_ug | 0.2520 | NS |
| Negative_Control vs CpG_50_ug | 0.2992 | NS |
| Negative_Control vs Positive Control | 0.0104 | S |
| 2X_CpG_50_ug vs CpG_100_ug | 0.0112 | S |
| 2X_CpG_50_ug vs CpG_50_ug | 0.1274 | NS |
| 2X_CpG_50_ug vs Positive Control | 0.1115 | NS |
| CpG_100_ug vs CpG_50_ug | 0.4901 | NS |
| CpG_100_ug vs Positive Control | 0.0010 | S |
| CpG_50_ug vs Positive Control | 0.0480 | S |

**Post-hoc comparisons of alpha diversity (Ace index) between treatment groups:** the first group (G1) was injected with 50 μg CpG intramuscularly (IM); the second group (G2) was injected with 100 μg CpG IM; the third group (G3) was injected with 50 μg CpG followed by a booster dose on day 22 post-hatch; and the fourth (G4; positive control) and fifth (G5; negative control) groups were injected with a saline solution. Birds in G1-4 were challenged on day 22 post-hatch with *C. perfringens*.
